# Meta-Analysis of Penetrance and Systematic Review on Transition to Disease in Genetic Hypertrophic Cardiomyopathy

**DOI:** 10.1161/CIRCULATIONAHA.123.065987

**Published:** 2023-11-06

**Authors:** Constantin-Cristian Topriceanu, Alexandre C. Pereira, James C. Moon, Gabriella Captur, Carolyn Y. Ho

**Affiliations:** Department of Medicine, Brigham and Women’s Hospital, Harvard Medical School, Boston, MA (C.-C.T., A.C.P., C.Y.H.). UCL Institute of Cardiovascular Science (C.-C.T., J.C.M., G.C.) and UCL MRC Unit for Lifelong Health and Ageing (G.C.), University College London, UK. Cardiac MRI Unit, Barts Heart Centre, West Smithfield, London, UK (C.-C.T., J.C.M.). The Royal Free Hospital, Centre for Inherited Heart Muscle Conditions, Cardiology Department, Hampstead, London, UK (G.C.).

**Keywords:** age of onset, cardiomyopathy, hypertrophic, penetrance

## Abstract

**BACKGROUND:**

Hypertrophic cardiomyopathy (HCM) is characterized by unexplained left ventricular hypertrophy and is classically caused by pathogenic or likely pathogenic variants (P/LP) in genes encoding sarcomere proteins. Not all subclinical variant carriers will manifest clinically overt disease because penetrance (proportion of sarcomere or sarcomere-related P/LP variant carriers who develop disease) is variable, age dependent, and not reliably predicted.

**METHODS:**

A systematic search of the literature was performed. We used random-effects generalized linear mixed model meta-analyses to contrast the cross-sectional prevalence and penetrance of sarcomere or sarcomere-related genes in 2 different contexts: clinically-based studies on patients and families with HCM versus population or community-based studies. Longitudinal family/clinical studies were additionally analyzed to investigate the rate of phenotypic conversion from subclinical to overt HCM during follow-up.

**RESULTS:**

In total, 455 full-text manuscripts and articles were assessed. In family/clinical studies, the prevalence of sarcomere variants in patients diagnosed with HCM was 34%. The penetrance across all genes in nonproband relatives carrying P/LP variants identified during cascade screening was 57% (95% CI, 52%–63%), and the mean age at HCM diagnosis was 38 years (95% CI, 36%–40%). Penetrance varied from ≈32% for *MYL3* (myosin light chain 3) to ≈55% for *MYBPC3* (myosin-binding protein C3), ≈60% for *TNNT2* (troponin T2) and *TNNI3* (troponin I3), and ≈65% for *MYH7* (myosin heavy chain 7). Population-based genetic studies demonstrate that P/LP sarcomere variants are present in the background population but at a low prevalence of <1%. The penetrance of HCM in incidentally identified P/LP variant carriers was also substantially lower at ≈11%, ranging from 0% in Atherosclerosis Risk in Communities to 18% in UK Biobank. In longitudinal family studies, the pooled phenotypic conversion across all genes was 15% over an average of ≈8 years of follow-up, starting from a mean of ≈16 years of age. However, short-term gene-specific phenotypic conversion varied between ≈12% for *MYBPC3* and ≈23% for *MYH7*.

**CONCLUSIONS:**

The penetrance of P/LP variants is highly variable and influenced by currently undefined and context-dependent genetic and environmental factors. Additional longitudinal studies are needed to improve our understanding of true lifetime penetrance in families and in the community and to identify drivers of the transition from subclinical to overt HCM.

**Clinical Perspective**

**What Is New?**

- In clinical studies on patients and families with hypertrophic cardiomyopathy, the prevalence of causal sarcomere or sarcomere-related variants was ≈34%; the penetrance of hypertrophic cardiomyopathy in relatives with pathogenic variants was ≈57%.
- In general population studies, the prevalence of pathogenic variants in sarcomere genes was 50-fold lower (0.7%), and the penetrance in those incidentally identified as variant carriers was 5-fold lower (11%).
- In longitudinal family studies, the pooled phenotypic conversion across all genes was ≈15% over an average of ≈8 years of follow-up, starting from a mean age of ≈16 years.

**What Are the Clinical Implications?**

- Because penetrance is context specific, different surveillance strategies may be appropriate for follow-up of at-risk family members compared with healthy individuals from the general population who are incidentally found to carry sarcomere variants.
- A multidisciplinary approach encompassing both basic and clinical investigation is needed to improve our understanding of penetrance of sarcomere variants and the transition from subclinical to clinically overt hypertrophic cardiomyopathy.

Multidisciplinary studies of patients and families with hypertrophic cardiomyopathy (HCM) have provided valuable insights establishing variants in genes encoding the sarcomere apparatus or sarcomere-related proteins as an important cause of HCM^1,2^ and highlighting the remarkable diversity and complexity of phenotypic manifestations, including age of onset, symptom burden, cardiac remodeling, prognosis, and even penetrance (proportion of variant carriers [G+] who develop left ventricular [LV] hypertrophy [LVH] or clinically overt HCM [G+LVH+]).^3^

Pathogenic or likely pathogenic (P/LP) variants associated with HCM are most commonly found in the core sarcomere genes, particularly myosin-binding protein C (MYBPC3; ~40% of sarcomeric HCM), β-myosin heavy chain (MYH7; 30%-40%), troponin T (TNNT2; 5%-10%) and troponin I (TNNI3; 5-10%) (Table 1). Genetic testing identifies a P/LP variant in ≈30% to 40% of all comers with a clinical diagnosis of HCM and in >60% in patients with familial disease.^4–7^ However, not all variant carriers manifest clinically overt HCM (G+LVH−, herein referred to as subclinical HCM), and in those who do, penetrance is age dependent, with disease typically developing during late adolescence through early to middle adulthood.^8,9^ Longitudinal studies have attempted to estimate the proportion of G+LVH− individuals who develop HCM and to estimate the rate of phenotypic conversion. However, individual studies have been limited by small size and short duration of follow-up.

**Table 1.**
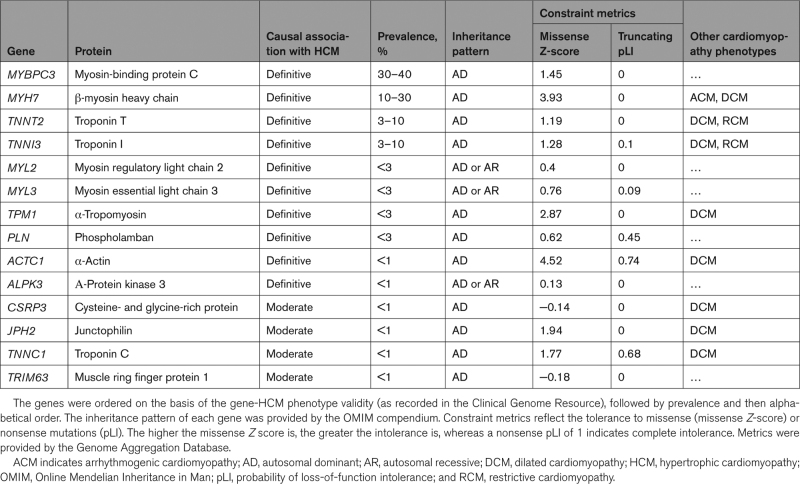
Genes Associated With HCM

Furthermore, the advent of unbiased genotyping in large-scale general population studies has led to the incidental identification of P/LP variants associated with HCM in individuals who typically do not have a known diagnosis. When cardiac imaging is also available, the percentage of variant carriers who have unexplained LV hypertrophy can be calculated to estimate penetrance. The penetrance and clinical impact of P/LP variants differ between family-/clinically-based studies and general populations studies, but these differences have not been systematically characterized.

To address these key questions, we performed meta-analyses to summarize the current understanding of the prevalence and penetrance of sarcomeric P/LP gene variants, comparing findings from family-/clinically-based studies with those reported in general population studies with accessible genetic and cardiac imaging data. Meta-analyses were additionally performed in longitudinal studies that followed up gene variant carriers to summarize the current knowledge of the transition from subclinical to clinically overt HCM.

## METHODS

### Compliance With Ethical Standards

The data used in this manuscript are publicly available, and ethics approval was not required.

### Search Process

The review was conducted to fulfill the PRISMA (Preferred Reporting Items for Systematic Reviews and Meta-Analysis) criteria on published peer-reviewed journal articles but also included preprint archives and conference proceedings full papers. Our review questions were the following: (1) What is the penetrance in cross-sectional studies and the phenotypic conversion in longitudinal studies of each sarcomere or sarcomere-related gene associated with HCM (as defined in Table 1)? (2) Does the penetrance differ between family- and clinically-based studies and population/community studies? (3) What is the age of HCM onset for each gene associated with HCM? (4) What is the prevalence of sarcomere or sarcomere-related P/LP variants in family and clinically-based HCM cohorts? (5) What proportion of asymptomatic individuals with unexplained LVH from the general population also carry a P/LP variant? (6) What is known about the transition from G+LVH− to G+LVH+? All English-language manuscripts and articles available online through electronic indexing addressing any of the review questions were included.

A systematic search of Embase, PubMed, Scopus, Google Scholar, MedRxiv, and BioRXiv was used to identify relevant manuscripts published up to March 1, 2023. Search items were defined with the PECO (patient/exposure/comparator/outcomes) framework: P=hypertrophic cardiomyopathy, HCM, LV hypertrophy; E=pre-phenotypic, prephenotypic, non-hypertrophic, nonhypertrophic, pre-LVH, pre-clinical, preclinical, sub-clinical, subclinical, early disease, gene carrier, mutation carrier, gene positive, gene mutation, HCM carrier; C=overt HCM, hypertrophic HCM, genotype positive HCM, phenotype positive HCM; and O=penetrance, phenotypic conversion, expressivity, age of onset, age of diagnosis, detection, prevalence, manifestations, phenotype, morphological, functional, dysfunction, and transition to disease. Instead of the generic word gene, individual genes from Table 1 were also included in the search queries. The PECO framework categories were combined using “and,” whereas we grouped the variations within categories by “or.” We excluded: (1) studies with <5 participants per gene, (2) studies exploring outcomes of interest in syndromic HCM or HCM phenocopies, (3) studies not using echocardiographic or cardiovascular magnetic resonance imaging (CMR)–based measurements of LV wall thickness to assess the presence of LVH, (4) studies that did not define or in which it was not possible to redefine overt HCM with the available data as a LV maximal wall thickness (MWT) ≥15 mm in probands and MWT ≥13 mm in relatives, (5) studies that did not use robust genotyping to assess mutation status, and (6) nonoriginal research (eg, reviews). If cohorts with overlapping membership were identified, the study with the highest sample size was included to minimize bias. As there are no validated tools to evaluate study quality within the specific scope of this review,^10^ a formal quality assessment was not pursued, but relevant studies were critically appraised.

### Statistical Methods

All statistical analyses were performed in R 4.2.1 with the packages meta and metafor. Random-effects (RE) generalized linear mixed models (GLMM) with binomial distribution and logit link were used to calculate the pooled penetrance (in cross-sectional studies), the phenotypic conversion (in longitudinal studies), and their associated 95% CIs for each HCM-related gene (Table 1) and across all genes.^11^ Because the studies included individuals of all age groups, including children and older adults, our penetrance estimates aim to provide a representation of the penetrance across the life course. The lack of individual participant data prevented the separation of age groups in most studies, leading to the inclusion of all age ranges in our analyses. In studies reporting only the number of included families, one proband per family was assumed to exist. In calculations of HCM penetrance, individuals who developed a different cardiomyopathy were excluded. Participants with multiple pathogenic variants were excluded if possible. With regard to the cross-sectional penetrance in family/clinical studies, we provide the penetrance in the relatives carrying P/LP variants identified as part of cascade screening. Studies in which the contribution of probands to the cross-sectional penetrance in relatives could not be separated were excluded from this analysis. For comparison, we also provide the cross-sectional penetrance across all carriers (ie, both probands and relatives). However, the later estimate is biased especially because the number of probands varied between the studies. With regard to the longitudinal phenotypic conversion, any participant with prevalent disease (regardless if proband or relative) was excluded. Studies in which the contribution of those with prevalent disease to phenotypic conversion could not be separated were excluded from this analysis. An RE meta-analysis model was used to calculate the pooled mean age at baseline and follow-up duration in longitudinal studies evaluating phenotypic conversion.

To calculate the mean age at HCM diagnosis across all genes and for each gene in all studies, an RE meta-analysis model was also used.^12^ In addition, a RE GLMM model was used to derive the prevalence of sarcomeric or sarcomeric-related P/LP gene variants in clinical HCM cohorts with ≥200 genotyped participants. We also report the prevalence in the subgroup that used the American College of Medical Genetics and Genomics criteria for variant classification. When calculating the mean age at HCM diagnosis or the prevalence of G+ in HCM cohorts, we included all carriers who had a confirmed diagnosis of HCM to minimize bias.

The reported penetrance, phenotypic conversion, age at HCM diagnosis, and prevalence of G+ in clinical HCM cohorts varied between the studies. However, some variation is expected to occur by chance (ie, random measurement errors). The heterogeneity between studies was appraised with the Cochran Q *P* value and Higgins *I*^2^ statistics. The 95% CI of *I*^2^ was also calculated. Either a Cochran Q *P*<0.05 or *I*^2^≥50% was interpreted as suggesting the presence of heterogeneity.

Meta-regression was used to study potential covariates that might influence penetrance when: (1) there was evidence of heterogeneity in the meta-analysis, (2) there were n≥5 studies per analysis, and (3) the covariate was reported by the studies included in the meta-analysis. In this study, it was feasible to explore age (as the mean age of the relatives), sex (as the percentage of male participants), and geography (the continent of the study participants) as covariates. In instances in which the origin of study participants was not explicitly stated, we used the continent corresponding to the senior author’s institutional affiliation. Studies spanning multiple continents were excluded from the geographic meta-regression. To visually depict trends, meta-regression scatter bubble plots were generated for continuous covariates and box plots for categorical ones.

Small-study effects is a phenomenon whereby studies with smaller sample sizes exhibit different (usually larger) effect sizes.^13^ The most common reasons are publication, selective outcome reporting, and confounding bias. For meta-analyses with ≥10 studies, the Egger test^14^ was used to assess for small-study effects, and values of *P*<0.05 were interpreted as potentially indicating their presence. Contour-enhanced funnel plots were also generated,^15^ and the presence of asymmetry was interpreted as indicating the possibility of publication bias, small-study effects, or methodological heterogeneity.

Last, we used a 2-sample *z* test for independent variables that takes into account variance to compare 2 estimates for which the 95% CIs were available.^16^

### Data and Code Availability

All the relevant data have been published in the article or the supplementary publication material. The generalized linear-mixed model meta-analysis code template can be accessed at https://cran.r-project.org/web/packages/metafor/metafor.pdf.

## RESULTS

Database searches identified 1734 articles. After the abstracts were screened, 455 full-text articles were assessed, and 115 met inclusion criteria for quantitative analysis. Figure S1 presents the PRISMA flowchart. Cross-sectional family or clinical studies that provided data on penetrance in proband relatives are presented in Table S1. Longitudinal studies exploring phenotypic conversion in G+LVH− are presented in Table S2. Studies providing only the age at HCM diagnosis per sarcomere or sarcomere-related gene in HCM cohorts are presented in Table S3. Studies with ≥200 genotyped participants providing the prevalence of gene variants in HCM clinical cohorts are presented in Table S4.

### Cross-Sectional Prevalence and Penetrance: Family and Clinical Studies

**Figure 1.**
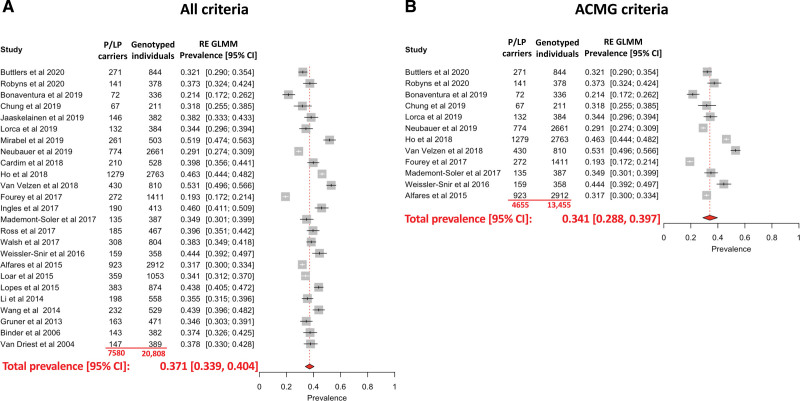
**Pooled prevalence of P/LP sarcomere or sarcomere-related gene variants in clinical HCM cohorts. A**, Prevalence in cohorts with ≥200 genotyped participants with hypertrophic cardiomyopathy (HCM), including probands. Smaller studies were excluded because they may introduce bias and potentially widen the CIs, affecting the precision of our results. **B**, Prevalence in studies that used the American College of Medical Genetics and Genomics (ACMG) criteria for variant classification. Neubauer et al^2^ 2019 used the Oxford Genetics Laboratory criteria, which closely follow the ACMG criteria, so that study was included in this analysis. GLMM indicates generalized linear mixed model; P/LP, pathogenic/likely pathogenic; and RE, random effects.

Figure 1A summarizes study-specific and pooled prevalence of sarcomere or sarcomere-related variants in clinical HCM cohorts, examining 20 808 participants from nonoverlapping cohorts with ≥200 genotyped participants. Using strict and standardized American College of Medical Genetics and Genomics criteria for variant classification, genetic testing identified a P/LP variant in 34% (95% CI, 29%–40%) of patients diagnosed with HCM (Figure 1B).

Focusing on family-based studies of kindreds with sarcomeric HCM, the pooled cross-sectional penetrance of all sarcomere or sarcomere-related gene variants in at-risk G+ relatives carrying the P/LP family variant (excluding probands) was 57% (95% CI, 52%–63%; Table 2). It is notable that penetrance differed from gene to gene (*I*^2^=55% [95% CI, 41%–65%]; Cochran Q *P*<0.001), ranging from 32% for myosin light chain 3 (*MYL3*) to ≈55% for *MYBPC3*, ≈60% for *TNNT2* and *TNNI3*, and ≈65% for *MYH7*. If probands were included, the penetrance was higher (*P*=0.013), reaching 67% (95% CI, 63%–72%) with similar gene-to-gene variation (Table S5). The mean age at diagnosis of HCM was 38 years (95% CI, 36–40) across all genes, but it varied from ≈33 years for *MYH7* to ≈41 years for *MYBPC3*.

**Table 2.**
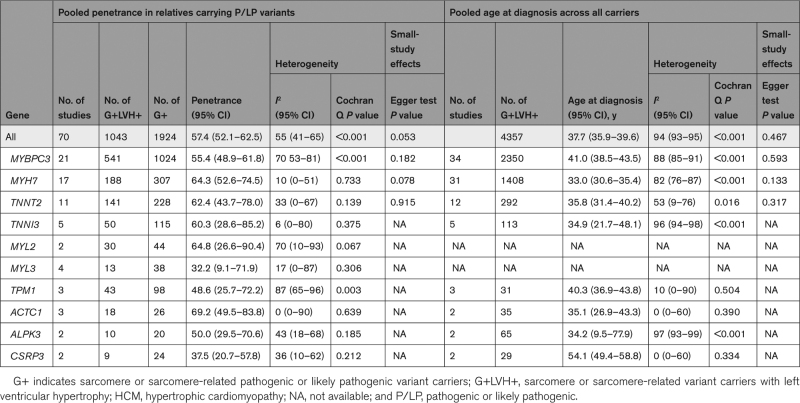
Penetrance and Age of Diagnosis in Familial HCM From Cross-Sectional Studies

**Figure 2.**
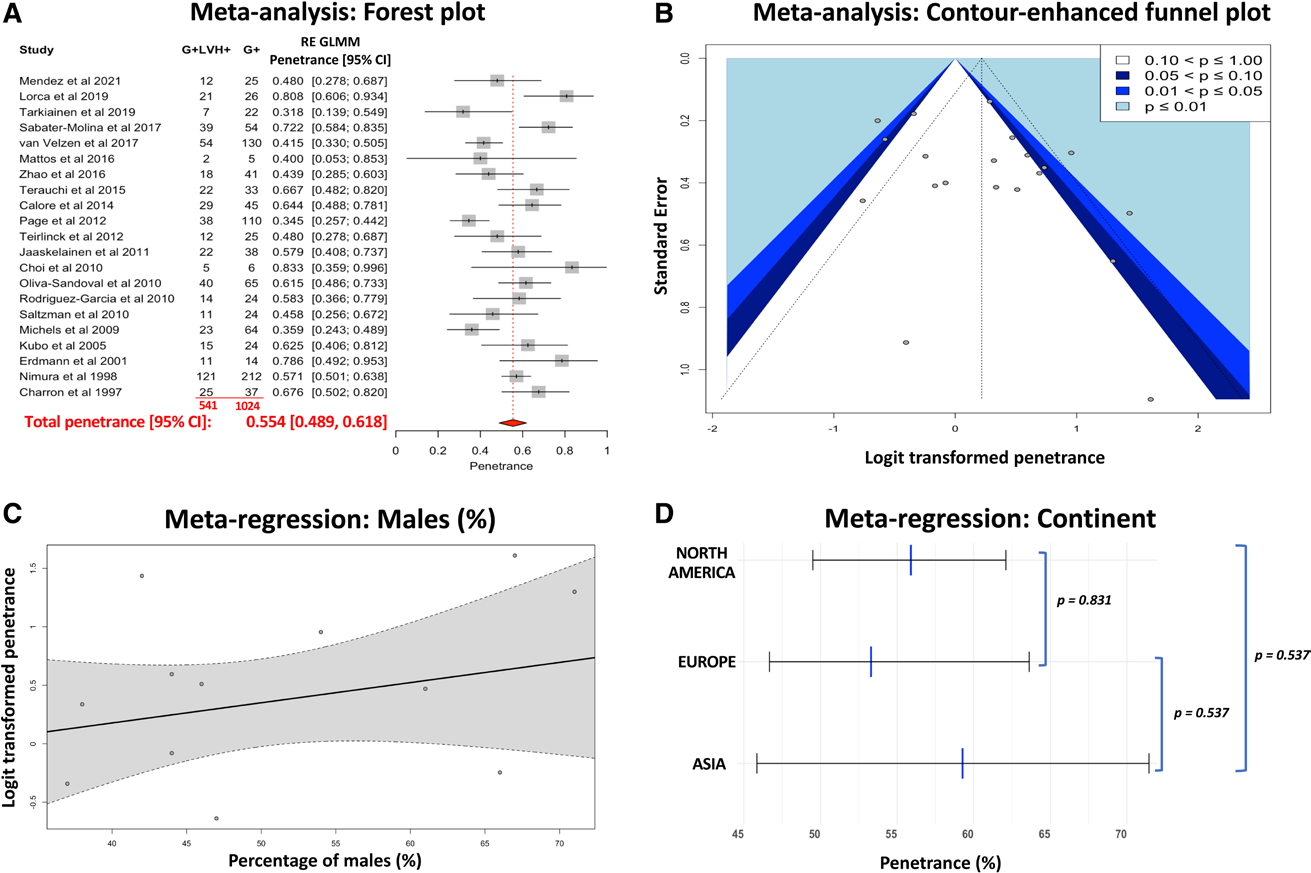
***MYBPC3* penetrance in cross-sectional family and clinically-based studies. A**, After probands were excluded, cross-sectional penetrance (defined as the percent of sarcomere or sarcomere-related pathogenic or likely pathogenic variant carriers [G+] with left ventricular hypertrophy [LVH]) was calculated using a random-effects generalized linear mixed model (RE GLMM) meta-analysis, and the corresponding forest plot is shown. Overall, the pooled penetrance in G+ relatives in families with hypertrophic cardiomyopathy who were identified as part of cascade screening was 55% (95% CI, 49%–62%). **B**, We explored whether this estimate could have been influenced by the tendency to publish only certain types of results (eg, reporting a very high penetrance). This is not supported by the contour-enhanced funnel plot, given its symmetry. **C**, We explored whether the penetrance is influenced by sex (using percentage of males as a covariate) and geography by study continent through meta-regression. Although the meta-regression bubble plot suggests that including more males was associated with reporting a higher penetrance, this association was not significant. **D**, The reported penetrance was 56% (95% CI, 50%–62%) in studies from North America, 55% (95% CI, 47%–64%) from Europe, and 59% (95% CI, 46%–71%) from Asia. *P* values for pairwise comparisons are provided and indicate similar estimates across these geographic regions. *MYBPC3* indicates myosin-binding protein C3.

**Figure 3.**
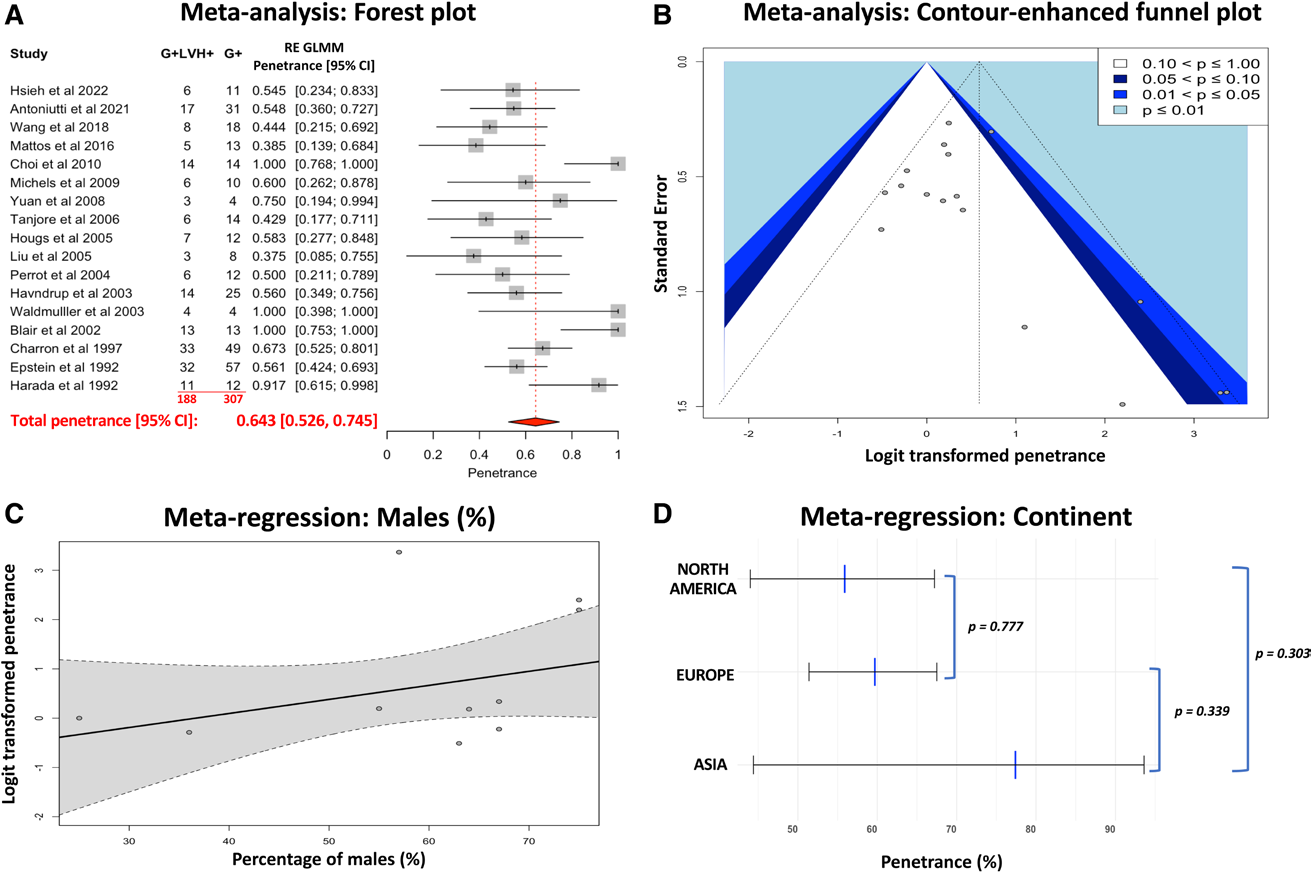
***MYH7* penetrance in cross-sectional family and clinically-based studies. A**, After probands were excluded, cross-sectional penetrance was calculated with a random-effects generalized linear mixed model (RE GLMM) meta-analysis, and the corresponding forest plot is shown. Overall, the pooled penetrance in relatives with sarcomere or sarcomere-related pathogenic or likely pathogenic variants (G+) in families with hypertrophic cardiomyopathy who were identified as part of cascade screening was 64% (95% CI, 53%–75%). **B**, We explored whether this estimate could have been influenced by the tendency to publish only certain types of results (eg, when reporting a very high penetrance). This is supported by the contour-enhanced funnel plot, given its asymmetry. **C**, We explored whether the penetrance is influenced by sex (using percentage of males as a covariate) and study continent through meta-regression. The meta-regression bubble plot does not suggest that including more males was associated with reporting a higher penetrance. **D**, The reported penetrance did not differ significantly by geography as represented by study continents, with values of *P*>0.05 for pairwise comparisons. LVH indicates left ventricular hypertrophy; and *MYH7*, myosin heavy chain 7.

*MYBPC3*, *MYH7*, and *TNNT2* were investigated further because they are the most prevalent genes associated with HCM. Results are presented in Table 2 and visually depicted in Figure 2 for *MYBPC3*, in Figure 3 for *MYH7*, and in Figure S2 for *TNNT2*. The pooled penetrance for the 1024 relatives carrying *MYBPC3* P/LP variants identified during cascade screening was 55% (95% CI, 49%–62%), and the mean age at HCM diagnosis was 41 years (95% CI, 39–44). However, the studies were heterogeneous, and penetrance ranged from <40% to >80% (Figure 2A). For *MYH7*, the pooled penetrance was 64% (95% CI, 53%–75%) across the 307 relatives with P/LP variants from the 17 family and clinically-based studies. Although the absolute estimate was higher, there was no statistically significant difference in penetrance between *MYBPC3* and *MYH7* (*P*=0.167). The *MYH7* studies were also heterogeneous, and penetrance ranged from ≈40% to 100% (Figure 3A). The mean age at HCM diagnosis in *MYH7* carriers was 33 years (95% CI, 31–35). In relatives carrying P/LP variants of *TNNT2*, the penetrance was 62% (95% CI, 44%–78%) at a mean age at diagnosis of 36 years (95% CI, 31–40), although the studies were heterogeneous, and penetrance ranged from 25% to 100% (Figure S2A).

There was no statistically significant difference in penetrance between *MYBPC3*, *MYH7*, *TNNT2*, *TNNI3*, *MYL2* (myosin light chain 2), and *TPM1* (tropomyosin 1; ≈50%–65%; all *P*>0.05). However, certain sarcomere or sarcomere-related genes may have a lower penetrance: ≈32% for *MYL3* and ≈38% for *CSRP3* (cysteine- and glycine-rich protein 3), although the sample sizes were smaller (ie, G+ n<50; Table 2).

To explore sex as a source of heterogeneity (ie, male and female participants having different penetrance rates or average age at HCM diagnosis), meta-regression was used. Sex-related differences in the overall or gene-specific penetrance estimates were not observed when the percentage of male participants was used as a covariate (Table S6). A 1% increase in the included percentage of males was associated with an increase of 0.2 years (95% CI, 0.1–0.3) in the mean age at HCM diagnosis across all genes (Table S6). We also evaluated whether studies in which the relatives had a higher mean age at the time of the study were more likely to report a higher penetrance. Fewer than half of the studies reported the age of relatives at the time of assessment, but according to available data, the mean age in relatives was 43.5±10.6 years across all studies and genes. With meta-regression, a 1-year increase in the age was associated with a 1% (95% CI, 0.1%–1.9%) increase in the reported penetrance (Table S7).

We also used meta-regression to explore whether the reported penetrance was influenced by geography. Across all genes, the highest observed penetrance was in studies conducted in Asia (68% [95% CI, 54%–79%]), followed by North American (62% [95% CI, 53%-70%]) and European (54% [95% CI, 48%–60%]) studies. However, only the difference between Asian and European studies reached statistical significance (*P*=0.047; Table S8).

### Cross-Sectional Prevalence and Penetrance: General Population Studies

Population studies with adequate genotypic and clinical data were analyzed to estimate the prevalence and penetrance of HCM in individuals incidentally discovered to be carrying P/LP sarcomere gene variants in the community. These studies included ARIC (Atherosclerosis Risk in Communities),^17^ FHS (Framingham Heart Study),^18^ JHS (Jackson Heart Study),^18^ and UK Biobank^19,20^ (Table 3). Across all cohorts, 1397 individuals carried a P/LP sarcomeric gene variant among the 213 911 genotyped participants. This indicates a pooled prevalence of 0.7% for P/LP sarcomere variants in the general population.

**Table 3.**
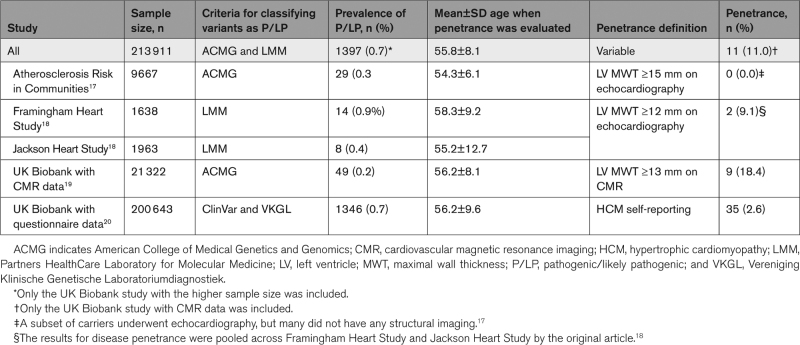
Prevalence and Penetrance in Large-Scale General Population Studies

The maximal LV wall thickness cutoff used to define LVH varied by study (MWT ≥12 mm in FHS and JHS, ≥13 mm in UK Biobank, and ≥15 mm in ARIC), and granular individual-level details on LV wall thickness measures are not available. When study definitions of LVH were used to indicate penetrance of HCM, the penetrance of P/LP sarcomere variants ranged from 0% in ARIC (none of the 29 P/LP variant carriers with a mean of 54±6 years of age had an MWT ≥15 mm on echocardiography, but only a subset had structural imaging^17^) to 18% in the UK Biobank (9 of 49 G+ participants with MWT ≥13 mm on CMR at a mean age of 56±8 years).^19^ The combined penetrance in FHS and JHS was 9% (2 of 22 G+ individuals aged 58±9 years on average with MWT ≥12 mm on echocardiography).^18^ Given the nonstandardized definitions of HCM, a meta-analysis was not formally performed. Overall, in the general population studies, the penetrance of sarcomere variants was 11%, at a mean age of 56±8 years.

### Longitudinal Family and Clinical Studies: Phenotypic Conversion From Subclinical to Clinical HCM

Longitudinal family and clinical studies were analyzed to estimate the incidence and rate of phenotypic conversion from subclinical to clinical HCM. This was performed for individual sarcomere genes and across all genes. Phenotypic conversion was defined as the percentage of G+LVH− individuals who developed overt HCM (G+LVH+) during follow-up. As summarized in Table 4, across all studies, 146 of 524 (28%) at-risk *MYBPC3*, *MYH7*, and *TNNT2* variant carriers developed HCM in studies in which a diagnosis was established during follow-up. However, there was substantial variability between studies, with Lorenzini et al^21^ reporting that 116 of their 226 *MYBPC3*, *MYH7*, and *TNNT2* carriers (51%) developed HCM (baseline age, 14±18 years; ≈15 years of follow-up), in contrast to only 30 of the 298 carriers (10%) combined in the remaining studies (mean baseline age, ≈16±11 years; ≈7 years of follow-up). To account for the difference in size and participant characteristics of the studies, we used an RE model to estimate that the pooled phenotypic conversion across these genes was 15% (95% CI, 8%–27%) starting from a mean age at baseline of 16 years (95% CI, 12%–20%) and during an average follow-up duration of 8 years (95% CI, 6–11), such that the mean age at the end of follow-up was ≈24 years (95% CI, 18–31). Results were heterogeneous (*I*^2^=81%; Cochran Q *P*<0.001), and the phenotypic conversion rate among individual studies ranged from 0% to 67%. As most longitudinal studies did not report the mean age at HCM diagnosis (Table S2), we were unable to perform a meta-analysis.

**Table 4.**
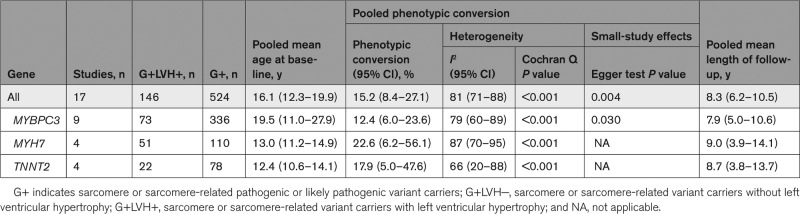
Phenotypic conversion of G+LVH− in Longitudinal Clinical Studies

**Figure 4.**
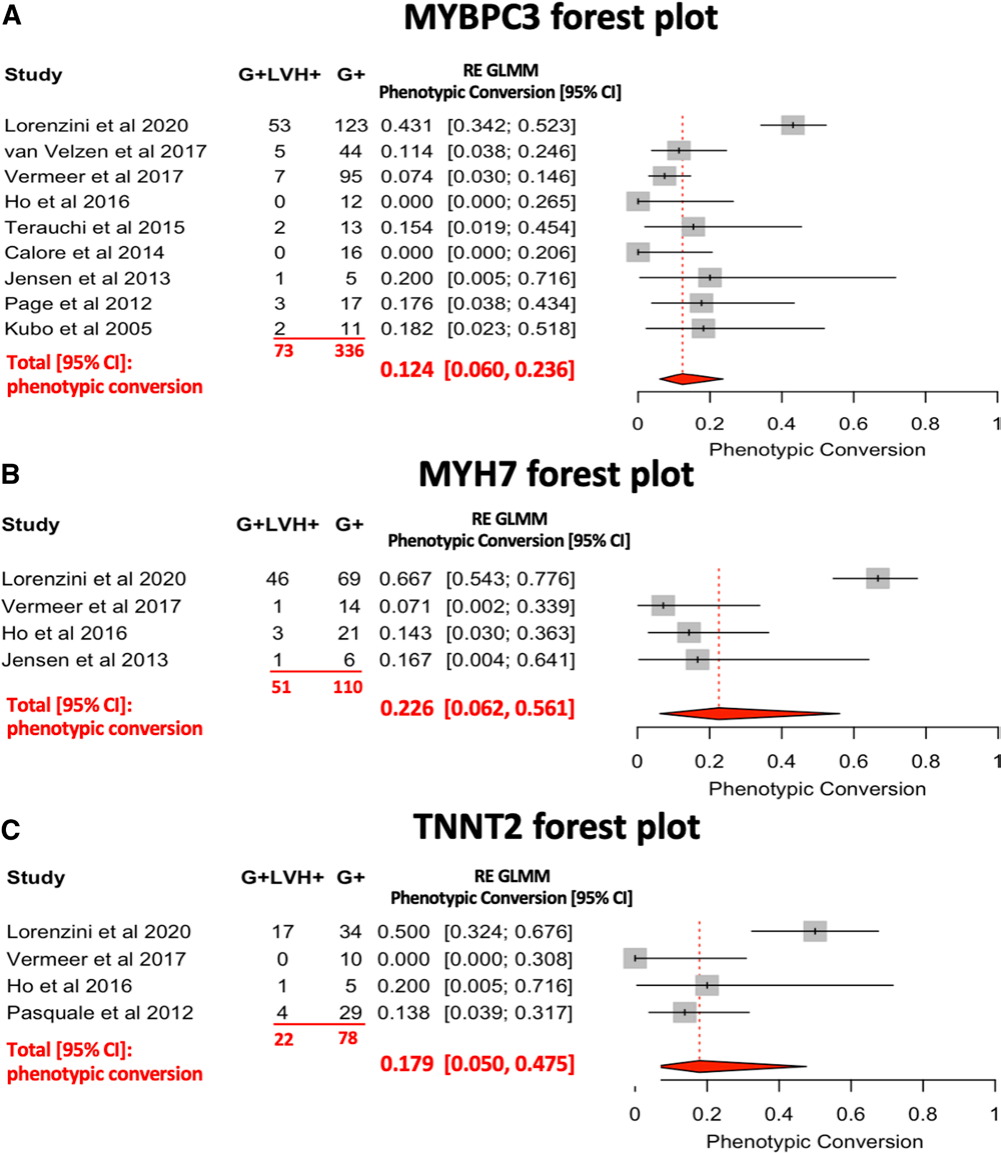
**Phenotypic conversion for *MYBPC3*, *MYH7*, and *TNNT2* genes in longitudinal studies.** Phenotypic conversion (defined as the percentage of sarcomere or sarcomere-related pathogenic or likely pathogenic variant carriers [G+] who developed left ventricular hypertrophy [LVH] during longitudinal follow-up) was calculated with a random-effects generalized linear mixed model (RE GLMM) meta-analysis. The forest plots for *MYBPC3* (**A**), *MYH7* (**B**), and *TNNT2* (**C**) genes are shown. Across all genes, the pooled phenotypic conversion was 15% over an average of ≈8 years of follow-up, starting from a mean age of ≈16 years. *MYBPC3* indicates myosin-binding protein C3; *MYH7*, myosin heavy chain 7; and *TNNT2*, troponin T2.

Analyzable studies reported gene-specific phenotypic conversion of *MYBPC3* (n=9), *MYH7* (n=4), and *TNNT2* (n=4; Table 4; Table S2). During a mean follow-up of ≈8 years starting from a baseline mean age of ≈20 years, 12% (95% CI, 6%–24%) of the *MYBPC3* variant carriers developed HCM. In contrast, 23% (95% CI, 6%–56%) of younger *MYH7* variant carriers (mean age, ≈13 years at initial evaluation) developed overt HCM over a similar follow-up period of ≈9 years. Phenotypic conversion in the 78 *TNNT2* variant carriers was 18% (95% CI, 5%–48%) during a mean follow-up period of ≈9 years, starting from an average age of ≈12 years. Lorenzini et al^21^ reported phenotypic conversion rates of >40% for *MYBPC3*, *TNNT2*, and *MYH7*. The higher rate of phenotypic conversion in their report was likely driven by the higher proportion of adults (≈40%), longer follow-up (≈15 years), and use of CMR to evaluate MWT, which may be more sensitive in detecting LVH than echocardiographic measures. Lorenzini et al^21^ additionally reported a phenotypic conversion of 17% (95% CI, 7%–39%) for *TNNI3* starting from a baseline age of 14 years during 15 years of follow-up, but a meta-analysis could not be conducted because of the absence of additional studies with data on this gene. The differences observed in the phenotypic conversion rates between genes were not statistically significant. Forest plots of the RE generalized linear-mixed model meta-analyses for *MYBPC3*, *MYH7*, and *TNNT2* are shown in Figure 4.

## DISCUSSION

**Figure 5.**
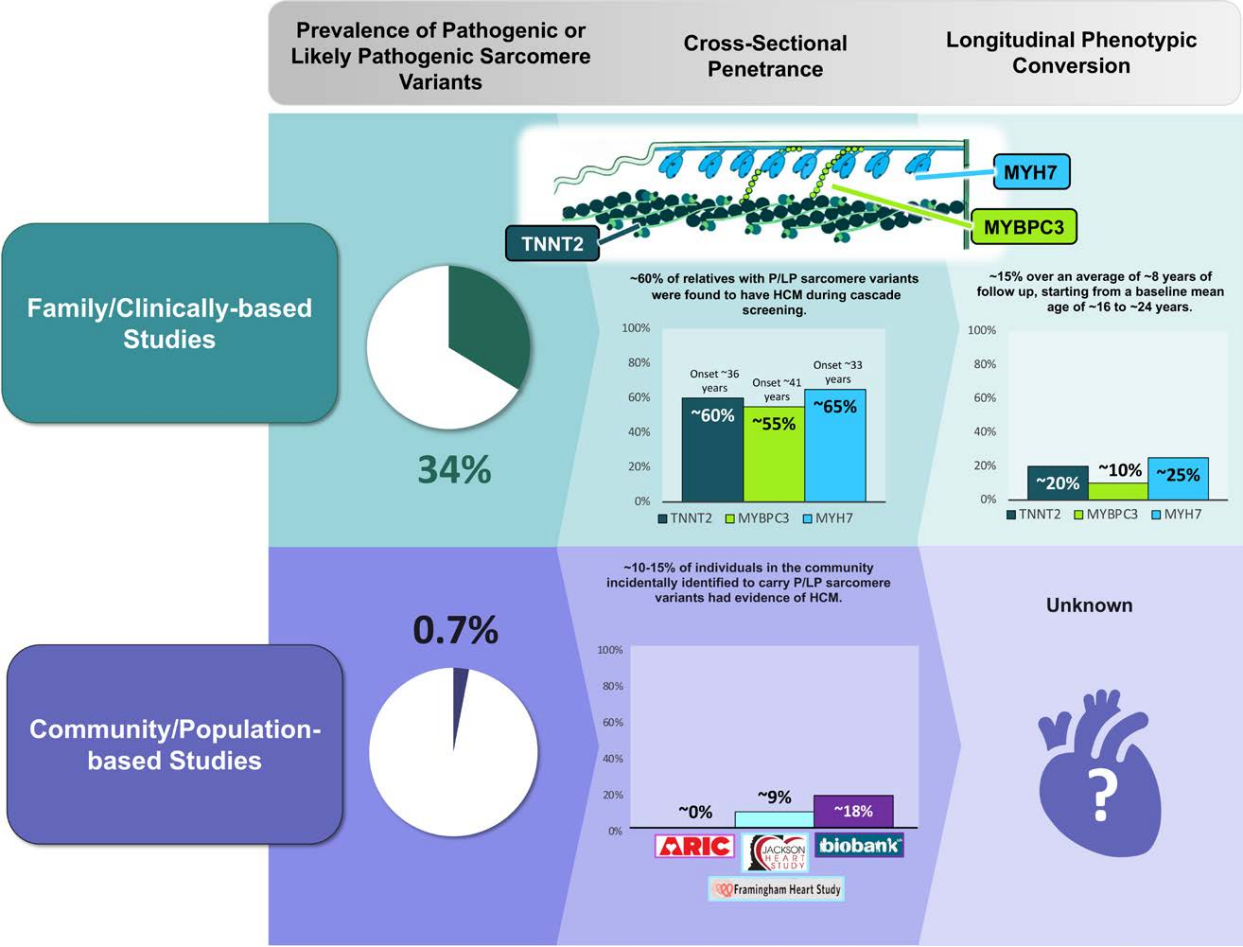
**Prevalence and penetrance of P/LP variants in family compared with population studies.** In clinical studies on patients and families with hypertrophic cardiomyopathy (HCM), the prevalence of pathogenic or likely pathogenic (P/LP) sarcomere variants was 34%, whereas the cross-sectional penetrance of HCM in relatives with P/LP was 57%. In contrast, in general population studies, the prevalence of P/LP variants in sarcomere genes was 50-fold lower (0.7%), and the cross-sectional penetrance in those incidentally identified as variant carriers was 5-fold lower (11%). In longitudinal family studies, the pooled phenotypic conversion across all genes was 15% over an average of ≈8 years of follow-up, starting from a mean age of ≈16 years. However, the phenotypic conversion in general population studies remains unknown. ARIC indicates Atherosclerosis Risk in Communities; *MYBPC3*, myosin-binding protein C3; *MYH7*, myosin heavy chain 7; and *TNNT2*, troponin T2.

In this study, we performed a meta-analysis to characterize the prevalence and penetrance of genetic variants that cause HCM in 2 different contexts: (1) clinically-based studies of families and patients with HCM, and (2) general population studies in which pathogenic variants were incidentally identified as part of unbiased genotyping. Findings are summarized in Figure 5. As expected, the prevalence of pathogenic or likely pathogenic variants in sarcomere or sarcomere-related genes was 50-fold higher and the penetrance was 5-fold higher in patients with HCM and relatives compared with sarcomere variant carriers incidentally identified in the general population. In phenotype-first clinically-based studies of ≈21 000 genotyped patients with HCM, the prevalence of P/LP sarcomere variants was 37% (34% when the more rigorous American College of Medical Genetics and Genomics criteria were used). Cross-sectional penetrance across all genes was 57% at a mean age of 44 years, with a mean age at diagnosis of 38 years. Penetrance ranged from ≈32% for *MYL3* to ≈55% for *MYBPC3*, ≈60% for *TNNT2* and *TNNI3*, and ≈65% for *MYH7*. Genotype-first population-based studies of ≈214 000 participants identified a low background prevalence of 0.7% for sarcomere variants in the general public. It is notable that applying a phenotype-first approach to population studies yielded a higher prevalence; ≈3% of the individuals with a CMR MWT ≥15 mm carried P/LP sarcomere variants in the UK Biobank.^19^ Despite meeting criteria for classification as P/LP, the penetrance of HCM was substantially lower (≈11%) when variants were identified incidentally in the general population, assessed at a mean age of 56 years across all studies. Penetrance was estimated at ≈11%, ranging from 0% in ARIC (assessed at 54±6 years of age) to 18% in the UK Biobank (assessed at 56±8 years of age).

When analyzing longitudinal family-based studies, only 15% of G+LVH− relatives developed HCM during ≈8 years of follow-up, starting at a mean age of ≈16 years. Short-term phenotypic conversion varied by gene, from ≈25% for *MYH7* to ≈20% for *TNNT2* and ≈10% for *MYBPC3*. Similarly, the age at HCM diagnosis varied by gene as carriers of *MYBPC3* variants tended to be expressed ≈10 years later than *MYH7*, *TNNT2* or *TNNI3* variant carriers. The meta-regression exploring the sources of heterogeneity suggested that studies including older carriers reported higher penetrance rates. This emphasizes the need for studies with extended follow-up of subclinical HCM, continuing through at least middle age, to more accurately estimate lifetime penetrance and phenotypic conversion given the wide variation in the age at which clinically overt disease develops. Indeed, an important limitation of these studies is that the duration of follow-up was relatively short and often ended before an age when features of clinically overt disease are most likely to emerge.

### Penetrance of P/LP Sarcomere Variants in HCM Cohorts Compared With the Community

Although present at a low level in the general population, the clinical impact of sarcomere variants classified as P/LP differed based on context. Penetrance for HCM was low in the general population; estimated to be 11%. Because the penetrance of sarcomere variants in HCM, like other adult-onset genetic disorders, is heavily influenced by age, reported cross-sectional penetrance underestimates true lifetime penetrance, as it merely captures the prevalence of the HCM phenotype at the time of the studies. However, assessments were performed at a mean age of 56±8 years, when sarcomeric HCM would be anticipated to have developed in most variant carriers. Indeed, studies in clinical cohorts reported a mean age of 30 to 35 years at diagnosis for *MYH7*, *TNNT2*, and *TNNI3*, and 40 to 45 years for *MYBPC3* and *TPM1*.

Sarcomere variants have been associated with subtle, often intranormal, abnormalities in LV wall thickness when healthy variant carriers in the general population were compared with noncarriers using machine learning approaches on CMR.^19,22^ We were not able to apply these analyses in this study. In addition, we did not assess the risk of heart failure and other adverse cardiac events in sarcomere variant carriers in the general population. Previous studies have indicated that adverse cardiac events were more prevalent in variant carriers than noncarriers in the population studies.^18^ These findings highlight the importance of developing more precise definitions of penetrance and clinical disease. Incidentally identified pathogenic variants in HCM-associated sarcomere genes may not be phenotypically silent even if criteria for HCM are not met. Although the anticipated penetrance of stereotypical HCM may be lower than in the family context, subtle abnormalities may result from sarcomere variants and may increase cardiac risk. Although careful longitudinal screening to monitor for evolution to HCM is critical for at-risk members of families with HCM, less intense follow-up for the emergence of HCM for individuals incidentally identified to carry P/LP sarcomere variants may be reasonable. However, it would be prudent to consider that they may be at heightened risk for developing other adverse cardiac events.

The difference in penetrance in population versus family studies emphasizes the presence of defined and undefined genetic and environmental factors associated with greater risk of developing HCM, including ethnicity,^23^ the presence of multiple P/LP variants,^24^ obesity,^25^ and hypertension.^26^ For example, the penetrance of HCM in *MYL3* carriers was 32%, contrasting with the 65% penetrance observed in *MYL2* carriers, although both genes serve a similar molecular function. Claes et al^27^ reported a penetrance of 89% in biallelic *MYL2* variant carriers or in heterozygous individuals suffering from hypertension or obesity versus 36% in *MYL2* variant carriers without additional risk factors. Propensity matching all participants for all genetic and environmental HCM risk factors was not possible in this study but would have provided some clarity on whether the gene-specific estimates are truly different. In addition, findings from large-scale genome-wide association studies in HCM suggest that genetic variants involved in myocardial growth, LV contractility, sarcomere organization, obesity, and blood pressure regulation could influence HCM susceptibility and even expressivity.^28–30^

### Phenotypic Conversion and Transition to Clinically Overt HCM

In longitudinal family studies with an average follow-up of ≈8 years, from a mean age of ≈16 to ≈24 years, the overall pooled phenotypic conversion was found to be 15%. There was gene-specific variation in short-term phenotypic conversion, ranging from ≈12% for *MYBPC3* to ≈18% for *TNNT2* and ≈23% for *MYH7*. However, differences in phenotypic conversion rates among specific genes were not found to be statistically significant. It is important to note that most longitudinal studies enrolled participants who were young at baseline, and the follow-up period concluded before participants reached the typical mean age of HCM onset. Thus, true penetrance and phenotypic conversion rates over a lifetime are underestimated.

The factors that drive either susceptibility or resilience to developing clinically penetrant HCM in individual sarcomere variant carriers are not understood. Previous studies have identified older age, male sex, and family history of HCM as risk factors for higher penetrance.^31–33^ A retrospective longitudinal study in 285 G+LVH− participants (from 156 families) suggested that an abnormal ECG quadrupled the risk of phenotypic conversion over ≈15 years, starting from a baseline average of 14 years of age.^21^ Another study suggested that progression to LVH in unrelated G+LVH− participants ≈16 years of age, followed up for a mean duration of 3 years, was more likely in those with longer mitral leaflets, lower global E’ velocities, and higher serum NT-proBNP (N-terminal pro-B-type natriuretic peptide).^34^ However, robust imaging and clinical biomarkers of impending progression of LVH and evolution to HCM are yet to be identified. Moreover, inflection points at which the natural course of the disease may be altered through interventions have not been elucidated.

### Limitations

An inherent limitation of our meta-analysis is the high heterogeneity of the included studies and their lack of external validation. The heterogeneity likely relates to study-to-study differences in the underlying prevalence of genetic and environmental risk factors associated with the development of HCM. We attempted to identify sources of heterogeneity that may influence the reported penetrance through meta-regression using study-level covariates such as the percentage of male participants or the age of the relatives. However, this approach is prone to ecological fallacy, as inferences made from group-level data might not accurately reflect individual-level relationships. In addition, the reported prevalence of P/LP variants is contingent on the criteria used for variant classification, which differed between studies and are somewhat subjective. This variability may affect population studies more than family studies in which rare sarcomere variants are more likely to be truly pathogenic. Although these differences in classification might bias our meta-analytic estimates, independently reclassifying all variants using the American College of Medical Genetics and Genomics criteria was not feasible because of the limited availability of variant data for individual participants. Most studies used echocardiography to measure LV wall thickness and to diagnose HCM. However, echocardiography may have a lower sensitivity in identifying LVH than CMR.^35^ We tried to obtain more accurate estimates by excluding participants who had multiple pathogenic variants, but most studies did not provide explicit information about the presence or absence of such complex genotypes in their participants. Last, we included only English-written manuscripts and articles available online, which could have biased our estimates and may decrease generalizability across different ancestries.

### Future Directions

A multidisciplinary approach integrating basic and clinical investigation is needed to improve our fundamental understanding of the penetrance and early phenotypic manifestations of sarcomere variants, as well as the transition from subclinical to clinically overt HCM. Long-term prospective study of subclinical HCM is specifically needed to provide: (1) better lifetime estimates of penetrance, (2) robust predictors that identify individuals most likely to develop penetrant disease, and (3) more accurate characterization of the full phenotypic spectrum of HCM, including changes leading up to and spanning the transition from subclinical to clinically overt HCM. Additional genome-wide association studies are also required to improve our understanding of genetic modifiers that convey susceptibility or resilience to developing phenotypic HCM. These insights will help guide management and surveillance decisions in the clinical arena, provide crucial insights to suggest novel therapeutic targets, and provide a means to assess the efficacy of novel therapies intended to modify disease progression or prevent disease emergence.

### Conclusions

The prevalence and penetrance of sarcomere variants differs according to the underlying context. Although variants classified as P/LP are present at a low level in the community, the likelihood of developing clinically overt HCM is 5-fold higher in studies on HCM cohorts and families compared with population studies. This highlights the presence of important but currently undefined genetic and nongenetic factors that influence the clinical impact of sarcomere variants. Because of the varying risk of developing HCM or other adverse cardiac outcomes, a more personalized approach to managing variant carriers is needed. Different strategies may be appropriate for members of families with HCM compared to healthy individuals who are incidentally found to carry sarcomere variants. Long-term prospective study of sarcomere variant carriers, in families and in the community, is needed to estimate the true lifetime penetrance and to improve our understanding of disease pathogenesis.

## ARTICLE INFORMATION

### Acknowledgments

The authors are grateful to Julia E. Marine for designing and creating Figure 5. All authors contributed significantly to the design, implementation, analysis, interpretation, and manuscript writing. The corresponding author attests that all listed authors meet the authorship criteria and that no others meeting the criteria have been omitted.

### Sources of Funding

Dr Ho is funded by the National Heart, Lung, and Blood Institute (P50HL112349 and 1U01HL117006). Dr Captur is supported by British Heart Foundation (MyoFit46 Special Programme Grant SP/20/2/34841) and the Barts Charity HeartOME1000 project grant (MGU0427/G-001411). Dr Moon is directly and indirectly supported by the UCL Hospitals NIHR BRC and Biomedical Research Unit at Barts Hospital, respectively. Dr Topriceanu is funded by the The Charlotte and Yule Bogue Research Fellowships in honour of Sir Charles Lovatt Evans and A.J. Clark. None of the funders was involved in the study design, data collection, analysis, or interpretation, or the decision to submit the manuscript for publication.

### Disclosures

The views expressed in this article are those of the authors, who declare that they have no conflict of interest, except for Dr Moon, who is the chief executive officer of Mycardium AI and has served on advisory boards for Genzyme and Sanofi.

### Supplemental Material

Tables S1–S8

Figures S1 and S2

References 36–146
